# Margin of Stability May Be Larger and Less Variable during Treadmill Walking Versus Overground

**DOI:** 10.3390/biomechanics1010009

**Published:** 2021-05-03

**Authors:** Farahnaz Fallahtafti, Arash Mohammadzadeh Gonabadi, Kaeli Samson, Jennifer M. Yentes

**Affiliations:** 1 Department of Biomechanics, University of Nebraska at Omaha; Omaha, NE 68182-0860, United States; 2 Rehabilitation Engineering Center, Institute for Rehabilitation Science and Engineering, Madonna Rehabilitation Hospitals; Lincoln, NE 68506, United States,; 3 Department of Biostatistics, University of Nebraska Medical Center; Omaha, NE 68198-4375, United States,; 4 VA Nebraska-Western Iowa Health Care System, Department of Veterans’ Affiars; Omaha, NE 68105, United States; 5 Department of Health & Kinesiology, Texas A&M University; College Station, TX 77843, United States

**Keywords:** extrapolated center of mass, mechanical gait stability, speed, heel contact, locomotion, walking

## Abstract

Margin of stability (MOS) is considered a measure of mechanical gait stability. Due to broad application of treadmills in gait assessment experiments, we aimed to determine if walking on a treadmill vs. overground would affect MOS during three speed-matched conditions. Eight healthy young participants walked on a treadmill and overground at Slow, Preferred, and Fast speed-matched conditions. The mean and variability (standard deviation) of the MOS in anterior-posterior and mediolateral directions at heel contact were calculated. Anterior-posterior and mediolateral mean MOS values decreased with increased speed for both overground and treadmill; although mediolateral mean MOS was always wider on the treadmill compared to overground. Due to lack of optic flow and different proprioceptive inputs during treadmill walking, subjects may employ strategies to increase their lateral stability on treadmill compared to overground. Anterior-posterior MOS variability increased with speed overground, while it did not change on treadmill, which might be due to the fixed speed of treadmill. Whereas, lateral variability on both treadmill and overground was U-shaped. Walking at preferred speed was less variable (may be interpreted as more stable) laterally, compared to fast and slow speeds. Caution should be given when interpreting MOS between modes and speeds of walking. As sagittal plane walking is functionally unstable, this raises the consideration as to the meaningfulness of using MOS as a global measure of gait stability in this direction.

## 1. Introduction

To maintain stability during walking (i.e., gait stability), the body’s center of mass (COM) must be controlled effectively with relation to the base of support provided by the feet. Ultimate lack of this mechanical gait stability is defined as a fall. Recovering from an unstable state by stepping in the forward direction is the essence of walking and progression in the anterior direction. In recent years, margin of stability (MOS) has been adopted to quantify mechanical stability while walking [1–4]. Combining both position and velocity of COM into a single outcome on a step-to-step basis is a key advantage of MOS compared to other stability measures. Moreover, the extrapolated COM concept can make predictions about foot placement including offset and proportional control [3]. The theory of extrapolated center of mass (X_coM_) is based on the linear inverted pendulum model, in which the projection of a point on the ground at a distance from the COM is proportional to the velocity of COM [5]. MOS was originally defined as the minimum distance between the extrapolated COM with respect to the base of support—as defined by the combined center of pressure—during walking in the medio-lateral (ML) direction [2,3]. Specifically, MOS was quantified when the distance between the extrapolated COM and base of support was shortest [2], typically occurring around contralateral toe-off. Recent studies have begun to calculate MOS in the anterior-posterior (AP) direction and at different phases of gait such as heel contact [6–10]. Heel contact is a key event during gait in which energy is absorbed. The position of the landing foot at heel contact determines foot placement during the stance phase, which is the dominant mechanism in the control of mechanical gait stability in the ML direction [11].

Decreased mechanical gait stability can be described by decreased mean or increased variability of MOS. Decreased MOS mean indicates less available space between extrapolated COM and boundaries of base of support; a more negative MOS indicates that the margin was more exceeded, and the extrapolated COM is outside of the base of support. In this situation, no margin is available to correct the exceeded distance of the extrapolated COM within the BOS boundaries. Moving the base of support, a stepping response, is the only option to stabilize as is demonstrated with increases in walking speed [12,13]. Moreover, MOS variability reveals information about step-to-step variations in the control of foot placement [6,14–16]. Increased MOS variability may be reflective of increased frequency of adjustments and corrective responses of foot placements in response to the environment or task demands [6,14].

In comparison with overground walking, when walking on a treadmill, preferred walking-speed can be slower, with shorter step lengths, and increased step frequency, sometimes referred to as a “cautious gait” on the treadmill [17,18]. When using this treadmill “cautious gait”, MOS in the ML direction was comparable to preferred walking-speed overground walking [19]; however, less is known about the comparison of MOS between two walking modes in the AP direction. Although, similar gait stability between treadmill and overground walking has not always been the case. Conflicting results of mechanical stability, i.e., MOS, during unperturbed treadmill versus overground walking, and across different walking speeds, have been reported [6,12]. Mechanical gait stability in the AP direction increased with faster speeds during treadmill walking [6,20], while a descending trend from slower to faster walking was observed during overground walking [1,7]. These reported differences may be due to differences in the calculation of MOS (not including the treadmill speed, which is the BOS velocity in [6,20]), methodology, and/or experimental protocols such as using kinetic vs. kinematic data for defining base of support and extrapolated COM [7,21], the mechanics of walking on two experimental conditions, or even the way extrapolated COM has been calculated during walking on treadmill compared to overground [4,6].

Therefore, the aim of this study was to determine if walking on a treadmill vs. overground would affect mechanical gait stability during three different steady-state speed conditions. Previously, different speeds at one walking mode (treadmill or overground) [6,12] or two walking modes at preferred speed were studied [19]. This study investigated the potential differences of walking mode during three matched walking speed conditions. Participants walked overground and on a treadmill at matched slow, preferred, and fast speeds. According to the previous studies using preferred walking speed with medium effect sizes [19,22], we hypothesized that the treadmill would not affect gait stability, meaning that there would be no significant difference for the main effect of walking mode (treadmill vs. overground) across all speeds. Our second hypothesis was that gait stability would be affected by speed. Walking with slower speed would provide greater gait stability compared to fast and preferred walking speeds.

## 2. Materials and Methods

### 2.1. Participants

Twelve healthy, young subjects (8 males, 4 females) aged between 19–35 years participated in this study. All subjects were physically active and without any neurological or musculoskeletal impairments. All subjects provided written informed consent for inclusion before they participated in the study. The study was conducted in accordance with the Declaration of Helsinki, and the protocol was approved by the Ethics Committee of (#435-18-EP). The University’s Institutional Review Board reviewed and approved all procedures.

### 2.2. Data Collection

Participants were asked to wear a form-fitting suit and fifteen retro-reflective markers were placed on defined anatomical locations of their feet and pelvis. Markers were placed on the toe, first and fifth metatarsal, posterior and lateral heel. Pelvis markers were located on the sacrum and the right and left anterior inferior iliac spine and posterior superior iliac spine. All participants were asked to walk on a treadmill at the pace they were comfortable walking casually to determine their self-selected walking speed (Preferred) following a previously described method [23]. As treadmill preferred walking speed has been shown to be slower than preferred overground speed [22], we selected treadmill preferred walking speed as the reference to not impose excessive effort during the fastest speed.

There was a total of six walking conditions (3 speeds × 2 walking modes). Two conditions at the participant’s preferred walking speed, two conditions at +40% of the participant’s preferred walking speed (Fast), and two conditions at −40% of the participant’s preferred walking speed (Slow) [24]. For each of the three speeds, one condition was on an instrumented treadmill (tandem force-sensing treadmill, AMTI, Watertown, MA, USA) and the other overground. Each treadmill trial lasted one minute, and the motion capture data recording was started after the treadmill had reached full speed and participants walked at least 10 steps on the treadmill. Overground, subjects walked back and forth over a single straight path, while speed was monitored simultaneously using the center of mass velocity (i.e., the average of right and left anterior and posterior superior iliac spines markers) through motion capture [6]. Overground speed at the Preferred, Fast, and Slow was to be within ±5% of their calculated treadmill speeds. To ensure at least 10 steps were collected for reliable variability analysis [25,26] from overground walking, a minimum of five trials per walking speed were collected. A minimum of two-minutes of rest was given periodically. Speed conditions were randomized within overground or treadmill. Kinematic data were collected from each trial at 120 Hz using motion capture (17 Raptor cameras, Motion Analysis Corp., Santa Rosa, CA, USA).

### 2.3. Data Analysis

**Figure 1.**
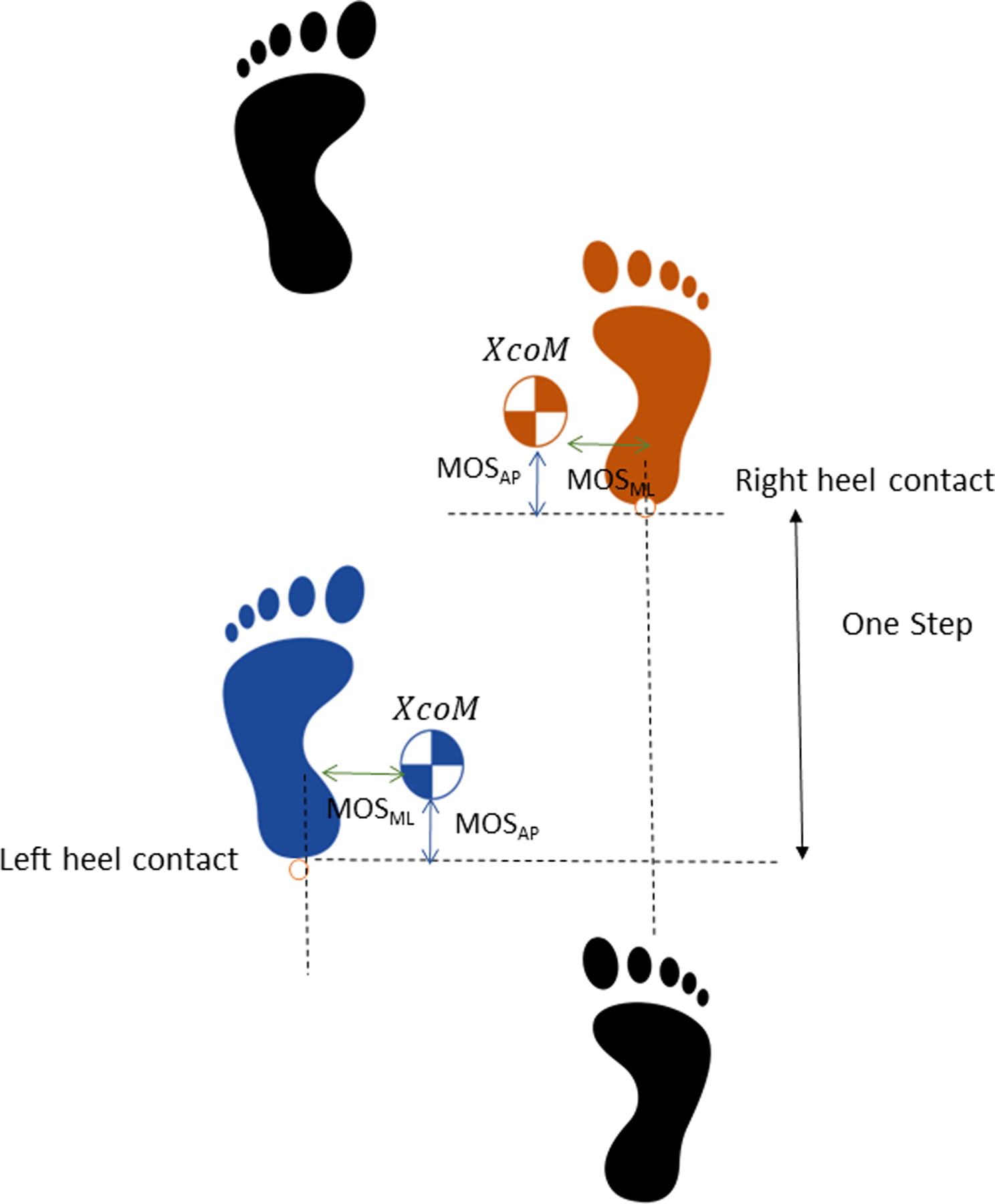
Defining corresponding steps and calculations. Steps were selected based on the speed criteria. Step speed was calculated as step length divided by step time. If a step was included for analysis, both the leading limb and trailing limb heel contacts from that step were used for the calculation of MOS. As can be seen in the figure, one step provided both a leading (orange) and trailing (blue) heel contact MOS in both the AP and the ML directions. The extrapolated COM was calculated at the time of the trailing limb heel contact and then again at the time of the leading limb heel contact. This is shown as two separate extrapolated COM positions in the figure. The MOS values were calculated for each heel contact with respect to the anterior limits of the base of support.

To determine which steps could be included in analysis from overground walking trials, the average center of mass velocity between two fixed (marked) points on the floor (~3 m distance between the points) in the middle of the walking path was calculated. When the average speed was within ±5% of their calculated treadmill speeds, these steps were included for analysis. All steps from the treadmill trials were eligible for analysis. Timing of heel contacts were determined using kinematics for all walking conditions [27]. Eighteen right and left heel contacts were extracted for MOS analysis, for a total of 36 events (Figure 1), from each of the six conditions. Mechanical gait stability was quantified at the heel contact event. Details of the calculation of MOS have been previously published [14]. Briefly, the extrapolated COM was calculated using this equation:

$$\text{XcoM }=\text{COM}+\frac{V_{\text{COM}}+V_{\text{BOS}}}{\omega_{0}}$$

where *V*_*COM*_ is the velocity of the center of mass and *V*_*BOS*_ is the velocity of the base of support. The position of COM was calculated based on average position of markers located on pelvis [21]. In this paper, during treadmill walking, the anterior velocity of the base of support due to the moving treadmill belt was added to the velocity of center of mass [14]; during overground walking, the velocity of the base of support was considered zero. $\omega_{0}$ is the eigen frequency of the pendulum defined as the square root of the acceleration due to gravity divided by the eigen frequency of the pendulum. The eigen frequency of the pendulum was defined in this work as the distance from the COM to the lateral right or left heel marker at heel contact [6]. The MOS was then calculated as the base of support minus the extrapolated COM. Base of support was defined using posterior heel marker position coordinates in the AP and ML directions, which is the projection of distal foot contact with the ground at this event. MOS was calculated in both the AP and ML directions at heel contact for each of the included 18 steps. MOS mean and variability (standard deviation) from each overground and treadmill speed condition was recorded.

For each of the 18 steps (Figure 1), the corresponding step speed, step length, step time, and step width were calculated. Step time and step length were determined as the time between the two contralateral heel contacts and the respective AP distance (during treadmill walking, the distance the belt moved between contralateral heel contacts was added to the distance). Step speed was calculated by dividing step length by step time for each step. Step width was the ML distance between the heel markers at consecutive heel contacts. The mean and variability (standard deviation) were calculated for each gait-related outcome during each condition. All calculations were performed using custom MATLAB programs (2018b; The MathWorks, Natick, MA, USA).

### 2.4. Statistics

Variables were inspected for normality and sphericity. Data were summarized using counts or means and standard deviations separately for participants. Differences in characteristics between conditions were assessed using *t*-tests. To identify the main effects of walking mode (treadmill vs. overground) and speed (Slow, Preferred, Fast), and the possible interaction between walking mode and speed, two different types of general estimating equations were used. One model type considered the mean/variability of all the steps per subject per speed per condition (i.e., each participant had six observations, one which summarized all steps (either mean or variability) for each combination of speed and condition; reported in the results section below). In the second model type (one model for right and one model for left limb), steps were entered as separate values per subject per speed per condition (i.e., each participant had 18 observations per combination of speed and condition; reported in the supplemental data). Significant interactions and main effects were further investigated with additional post-hoc comparisons, accompanied by simulated adjusted *p*-values to account for multiple comparisons [28]. Findings regarding the main effect of condition were confirmed using Bayesian paired-sample *T*-tests. Results of these tests are reported in Supplemental 2.

To identify potential confounding variables, spatiotemporal means and variability (i.e., step speed, step length, step time, and step width) were correlated with MOS means and variabilities in both the AP and ML directions. Spatiotemporal variables that demonstrated a moderate to strong relationship (r > 0.3) with MOS were included as covariates. All models were adjusted for preferred walking speed (m/s). Based on the correlation analysis, ML mean models were additionally adjusted for step width, however no additional adjustment variables were included in the AP mean model. AP variability models were additionally adjusted for step speed variability. ML variability models were additionally adjusted for step width variability. Effect sizes (ES) were calculated by dividing the model estimated mean difference by the standard error associated with the mean difference; 95% confidence intervals were also calculated and reported. All analyses were performed using SAS (version 9.4, SAS Institute Inc., Cary, NC, USA) or SPSS (version 23, IBM Corp., Armonk, NY, USA). The significance threshold was α = 0.05.

## 3. Results

Four subjects were excluded from the final analysis due to insufficient number of speed-matched steps across modes of walking. Therefore, data of the remaining eight subjects were included in the analysis (Table 1).

**Figure 2.**
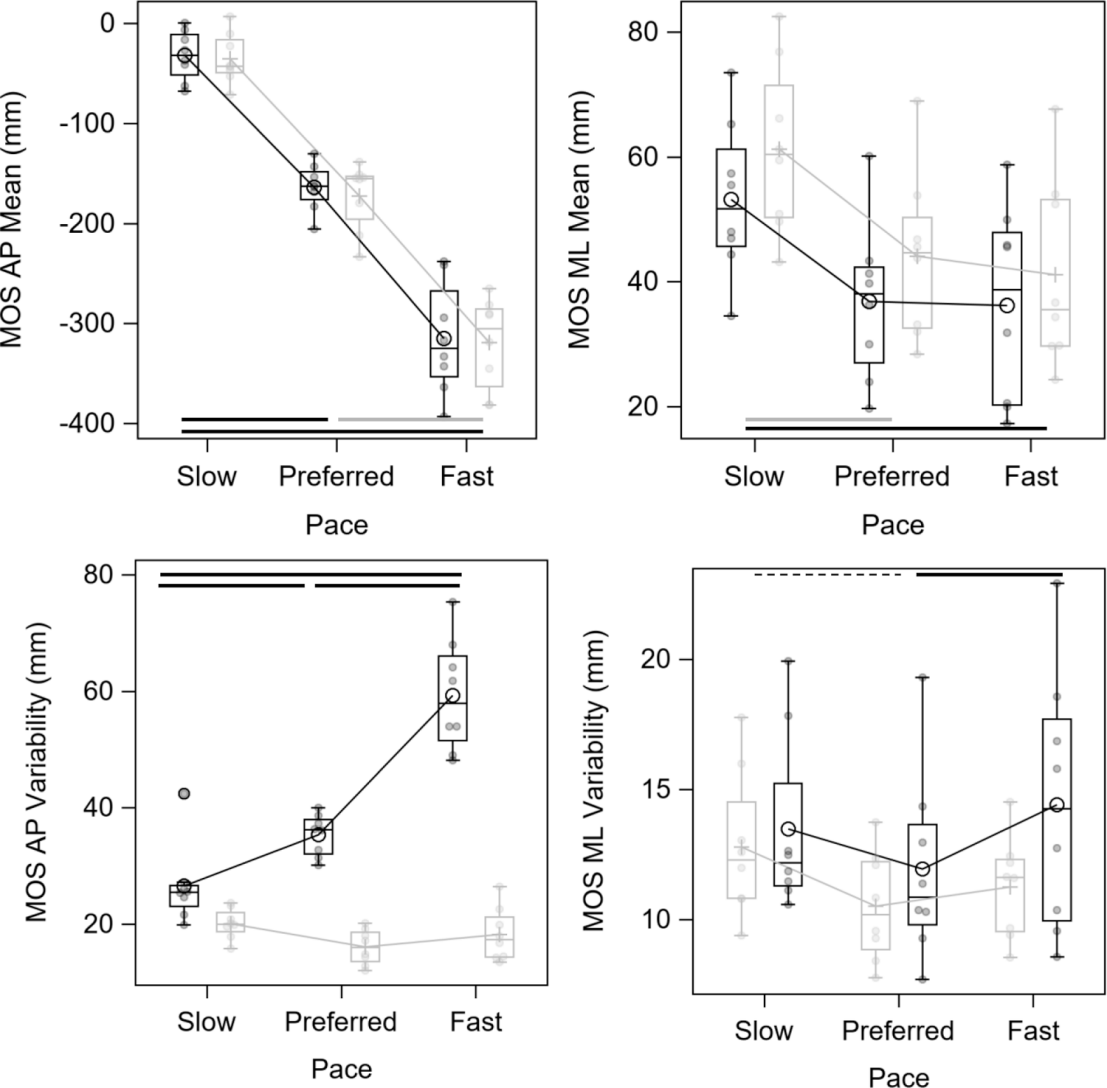
Anteroposterior (AP; left column) and mediolateral (ML; right column) margin of stability (MOS) actual means (top row) and variability (bottom row) for both modes treadmill (gray) and overground (black) and each walking speeds at heel contact (figures are from raw data). NOTE: Solid black horizontal bars represent the main effect of speed across both treadmill and overground conditions. Solid gray and dotted black horizontal bars represent the significant speed differences across treadmill and overground conditions (interaction), respectively.

MOS ML mean during treadmill walking was significantly larger than overground across all walking speeds with a large effect (*p* = 0.01; ES = −3.89) (Figure 2). MOS ML mean during Fast and Preferred was significantly smaller compared to Slow across both modes of walking (*p*’s < 0.0001; ES range = −6.05 to −1.96). No interaction was found for MOS ML mean. A significant interaction of speed condition and walking mode was found for MOS ML variability (*p* = 0.03). Post hoc tests revealed significantly increased variability during Slow relative to Preferred for the treadmill mode (*p* < 0.05). For overground, significantly increased variability during Fast relative to Preferred was found (*p* < 0.05).

No significant effect of walking mode was found for MOS AP mean (*p* = 0.89; ES = −0.14) nor interaction (*p* = 0.65). Walking faster led to a more negative MOS AP mean in both walking modes (*p* < 0.0001, all speeds were significantly different from each other) with ES ranging from −20.52 to −17.55. (Table 2). This large effect indicated that the extrapolated COM was further anterior to the base of support as they walked faster; thereby, MOS AP mean become more negative in faster speeds (Figure 2). There was a significant interaction between speed and walking mode for MOS AP variability (*p* < 0.0001). When walking overground, variability increased as subjects walked faster (*p*’s < 0.0001). In contrast, variability did not change with speed when walking on the treadmill (Table 3).

## 4. Discussion

This study investigated if treadmill walking would affect MOS at three different speeds compared to overground at heel contact. Contrary to our first hypothesis, mechanical gait stability in the ML direction was larger during treadmill walking compared to overground (Supplemental Data 2). There was no difference in mean stability margins between walking modes in the AP direction (Supplemental Data 2). Our second hypothesis speculated that walking slower would be more stable. In agreement with our hypothesis, we observed a large effect that the extrapolated COM was further anterior to the base of support as participants walked faster. This was the case during overground and on the treadmill. Similarly, in the ML direction, margins of stability narrowed as speed increased. Furthermore, variability in the AP directions increased with speed while walking overground but remained unchanged on the treadmill. Whereas, on the treadmill and overground, lateral variability may have been U-shaped, in which Preferred was less variable laterally compared to Fast and Slow.

Participants had less lateral mechanical gait stability when walking overground as the mean lateral MOS was narrower and the variability was increased compared to walking on the treadmill. Participants may try to increase lateral stability on the treadmill by voluntarily increasing base of support via control of foot placement. When walking on a treadmill, subjects may employ strategies to increase stability due to perceptual differences between treadmill and overground walking. For example, during treadmill walking the absence of natural optic flow decreases sensory information, which is more heavily weighted for control of movement in the ML direction [29], as well as different proprioceptive input (legs being pulled back during stance phase on the treadmill while remain stationary during overground). Wider MOS mean while walking on the treadmill compared to overground could be a potential factor restricting MOS variability during treadmill walking compared to overground.

Moreover, lateral stability was increased during the slowest speed of walking in both walking modes. During Slow additional balance challenges may be present due to increased ML COM motion [30], which requires compensatory increases of the MOS adjustments. Slower walking is characterized by increasing stride frequency and decreasing the stride length, which has been shown to increase mechanical gait stability in the ML direction in an effort to reduce the risk of fall [31,32]. A previous report has shown an increase in ML MOS with faster walking speeds, opposite of what is reported here. It is important to note that Burke et al. (2019) [33] calculated the MOS at contralateral toe-off, not necessarily at a particular point in the gait cycle such as heel contact. Although they found no relationship between step width and MOS during symmetric gait, Rosenblatt et al. (2012) reported that step width is chosen to maintain a minimum MOS [34]. Thus, it is plausible that step width is a confounding variable when calculating ML MOS. In the current study, a relationship between step width and ML MOS was found and, therefore, step width was entered into the statistical models as a covariate. This could likely be an additional reason as to why the current results differ.

Variability of lateral mechanical gait stability may have a U-shaped relationship for both walking modes. Specifically, compared to Preferred, Fast had increased variability when walking overground; and when walking on the treadmill, slow had increased variability compared to Preferred. In both walking modes, Preferred demonstrated optimal variability, which is in line with the previous studies indicating the U-shaped relationship of gait variability and speed in healthy young adults [35]. According to the dynamical system theory, walking at Preferred is a stable attractor state, and moving from this state may lead to instability and increase variability [36].

The results show that the small effect in AP mean gait stability was not different between the two walking modes. About 68% of extrapolated COM motion can be predicted using the step width and length from the current and four preceding steps [37]. Murray et al. reported that during non-matched slow, preferred, and fast treadmill walking, subjects walked with shorter step lengths compared to overground [18]. However, considering the increased step width during treadmill walking, there could be a trade off in the control of COM movement for the adaptation of stability, which may have led to no significant difference between mechanical gait stability in the AP direction between the two walking modes. However, walking faster led to more negative margin of stability in the AP direction for both walking modes, with very large differences, due to the extrapolated COM being located further outside the anterior boundaries of base of support. During faster walking, the whole-body momentum, which acts through passive dynamics, drives the “fall forward” gait mechanism, which leads to stepping and thus continued walking. An alternative explanation arises from the dynamics of the COM in the AP direction are governed more so by mechanics as compared to ML direction [29]. Due to these mechanics, the extrapolated COM continuously exceeds the BOS in the AP direction as walking is inherently unstable in the AP direction. It is unclear if the MOS in the AP direction can be used as an overall indicator of gait stability. Meaningfulness of the interpretation of MOS in the AP direction should be more carefully considered in future research as it may not capture overall gait stability.

MOS variability in the AP direction when walking overground was not mimicked on the treadmill. We observed increased variability of overground mechanical gait stability at faster speeds in the AP direction, yet no difference across speeds on the treadmill. Greater stride length variability during overground compared to treadmill (Table 1 and [29]) could be a potential factor for increased variations of gait stability in the sagittal plane during faster speeds, which are more demanding than slower speeds. Stride length control is speed dependent and mechanically driven, rather than being controlled by sensory information [38]. Although speed is fluctuating constantly during walking, participants maintain the preferred step length [38]. In the absence of an external speed cue as on a treadmill, overground selection of step length depends on different factors such as optic flow, time constraint, push-off [39], and energy efficiency [40], which could be underlying reasons for the difference in mechanical gait stability variability between the two walking modes. Walking with any change in step length can change the MOS in the AP direction [41]. On the treadmill, due to the belt moving, participants were not able to walk slower during an overall average step than the designated speed of belt (it may have led to instability) (see Supplemental Data 1). This may be considered a constraint imposed by the treadmill, while overground walking provided the freedom for participants to fluctuate their speed, which might be the reason for this difference in mechanical gait stability variability. During Slow, this difference between two walking modes was not observed, which could be due to different mechanical characteristics of slow speed such as reduced kinematic range of motion [42] and increased stance time (longer stable duration) compared to Preferred and Fast [43,44].

### Limitations and Conclusions

Using the preferred walking speed on the treadmill as the reference to match the speed between two modes was a limitation of this study, as treadmill preferred walking speed has been shown to be slower than preferred overground speed [22]. However, we selected treadmill preferred walking speed as the reference to avoid extra exertion during the fastest condition. Moreover, the inherent features of treadmill walking such as the absence of optic flow and different mechanical compliance of surface may affect the gait parameters. MOS is an instantaneous measure at one time point. Whether or not the MOS value indicates “general” gait stability or instability is highly context dependent. We have narrowed the description of MOS to heel contact event, and other gait events can be studied in the future. Finally, for calculation of stability margins, various suggestions have been proposed to define base of support limits during walking. It is important to discriminate between the base of support, where the boundaries located, and the effective base of support, the area in which the initial extrapolated center of mass should be located so that balance can be restored. This effective base of support is usually smaller than the foot boundaries, which is commonly supposed and is yet to be determined [45]. These general limitations across many MOS studies may make between-study comparisons more difficult. Lastly, it must be considered that only eight subjects were used for analysis. Resource limitations restricted the ability to include additional subjects in the study to add to the sample size. As a result, the study is only powered to detect very large differences and cannot make any conclusions about small or medium differences.

## 5. Conclusions

In conclusion, walking mode affects stability. Lateral stability was increased when walking on the treadmill compared to overground. On the treadmill and overground lateral variability was U-shaped, which indicated walking on preferred speed is less variable (can be interpreted as more stable) laterally, compared to Fast and Slow. Variability of sagittal stability differed between the two walking modes. Sagittal variability increased with speed when walking overground, while on the treadmill, it did not differ with speed. Mean sagittal stability did not differ across conditions; this is likely due to passive mechanical control in the AP direction. Further, in the AP direction, functionally, the center of mass exceeds the boundaries of the base of support to propel the body forward, i.e., falling forward description of walking. As sagittal plane walking is functionally unstable, this raises the consideration as to the meaningfulness of using MOS measures in the anterior posterior direction. Comparison of treadmill-based margin of stability means and variability should not be extended to that of overground walking, particularly at higher speeds of walking.

## Supplementary Material

Supplemental 2

Supplemental 1

## Figures and Tables

**Table 1.** Demographic data for the participants (n = 8; females = 3) used in the analysis. All values are written as mean (standard deviation).

Age (years)	23 (2.39)				
Body mass (kg)	72.42 (12.90)				
Height (cm)	175.75 (5.84)				
Preferred speed (m/s)	1.3 (0.09)				
	Treadmill	Overground	*p*−Value	95% Confidence Interval of the paired differences	
Step width (mm)				Lower	Upper
Slow	145.78 (30.79)	137.81 (37.24)	0.28	−8.25	24.18
Preferred	132.89 (36.39)	130.22 (33.68)	0.70	−12.96	18.30
Fast	136.38 (39.32)	140.41 (33.64)	0.45	−16.02	7.96
Step time (s)					
Slow	0.69 (0.03)	0.69 (0.03)	0.80	−0.03	0.03
Preferred	0.53 (0.03)	0.54 (0.03)	0.16	−0.015	0.01
Fast	0.46 (0.03)	0.47 (0.03)	0.53	−0.01	0.01
Step length (mm)					
Slow	559.52 (38.94)	538.87(33.31)	0.05	0.20	41.11
Preferred	716.62 (50.79)	708.58 (49.15)	0.14	−3.43	19.51
Fast	869.79(64.96)	857.56 (65.00)	0.12	−3.94	28.40
Step speed (mm/s)					
Slow	811.18 (57.50)	778.84 (54.81)	0.001	17.64	47.04
Preferred	1339.81 (101.43)	1312.28 (107.67)	0.01	8.72	46.36
Fast	1866.74 (136.02)	1830.67 (150.91)	0.004	15.41	56.74
Step width variability (mm)					
Slow	16.56 (4.06)	20.23 (7.76)	0.24	−10.46	3.12
Preferred	17.23 (2.87)	24.72 (4.18)	0.01	−12.65	−2.33
Fast	18.98 (3.00)	28.41 (12.41)	0.06	−19.19	0.33
Step time variability (ms)					
Slow	23.25 (6.28)	25.92 (5.51)	0.48	−0.01	0.01
Preferred	9.84 (2.61)	13.56 (2.83)	0.03	−0.01	−0.001
Fast	8.79 (0.99)	13.09 (3.06)	0.004	−0.01	−0.001
Step length variability (mm)					
Slow	24.78 (6.91)	25.57 (5.47)	0.81	−8.26	6.68
Preferred	15.55 (3.75)	21.01 (5.85)	0.03	−10.33	−0.58
Fast	18.27 (1.58)	24.18 (5.43)	0.01	−10.21	−1.62
Step speed variability (mm/s)					
Slow	20.26 (5.99)	45.46 (8.17)	<0.0001	−33.52	−16.88
Preferred	19.32 (5.19)	54.38 (17.22)	<0.0001	−48.20	−21.92
Fast	22.71 (3.4)	64.38 (15.71)	<0.0001	−54.34	−29.02

**Table 2.** Model Estimated Means from Linear Mixed Models of mean margin of stability (MOS) in the anterior posterior (AP) and mediolateral (ML) directions. No significant interactions were found, therefore main effect and post hocs are presented in the table. Each model adjusts for preferred walking speed. The ML Mean model additionally adjusts for step width.

	Pace and Condition Subgroups	Model Estimated Mean	Standard Error	95% Confidence Interval for Estimated Mean		Significant Post Hocs	*p*-Value for Interaction	Group Comparison for Effect Size	Effect Size
MOS ML Mean							0.78		
	Pace:					<0.0001^††^			
	Fast	38.32	2.98	31.92	44.72			F vs. P	−1.96
	Preferred	42.36	1.68	38.75	45.97			P vs. S	−6.05
	Slow	55.73	2.54	50.29	61.17			F vs. S	−4.54
	Condition:					0.01			
	Overground	42.16	2.16	37.04	47.27			O vs. T	−3.89
	Treadmill	48.78	1.89	44.31	53.26				
MOS AP Mean							0.65		
	Pace:					<0.0001^†^			
	Fast	−317.02	10.76	−340.10	−293.93			F vs. P	−17.94
	Preferred	−167.88	6.61	−182.07	−153.69			P vs. S	−17.55
	Slow	−33.58	7.89	−50.49	−16.66			F vs. S	−20.52
	Condition:					0.89			
	Overground	−172.23	7.56	−190.11	−154.34			O vs. T	0.14
	Treadmill	−173.42	7.56	−191.31	−155.54				

† All pace conditions significantly differ from each other (*p* < 0.0001).

†† The slow condition is significantly greater than preferred (*p* < 0.0001) and fast (*p* = 0.001) conditions.

**Table 3.** Model Estimated Means from Linear Mixed Models of margin of stability (MOS) variability for anterior posterior (AP) and mediolateral (ML) directions. Significant interactions were found, and comparisons were made between speeds, separately for each walking condition. Each model adjusted for preferred walking speed. Step speed variability and step width variability were additionally adjusted for in the AP and ML models, respectively.

	Pace and Condition Subgroups	Model Estimated Mean	Standard Error	95% Confidence Interval for Estimated Mean		Significant Post Hocs	*p*-Value for Interaction	GroupComparison for Effect Size	Effect Size
MOS ML Variability							<0.03		
	Overground:								
	Fast	14.68	1.41	11.65	17.71	*		F vs. P	3.56
	Preferred	12.10	1.27	9.37	14.82			P vs. S	−1.35
	Slow	13.48	1.32	10.65	16.32			F vs. S	0.94
	Treadmill:								
	Fast	11.17	0.83	9.38	12.96			F vs. P	1.84
	Preferred	10.37	0.78	8.69	12.05			P vs. S	−3.73
	Slow	12.64	0.83	10.87	14.41	*		F vs. S	−1.99
MOS AP Variability							<0.0001		
	Overground:								
	Fast	55.59	3.46	48.17	63.02	**		F vs. P	9.58
	Preferred	33.02	1.69	29.40	36.64			P vs. S	3.19
	Slow	25.52	2.31	20.57	30.47	* ^,†^		F vs. S	9.37
	Treadmill:								
	Fast	20.39	2.18	15.72	25.05			F vs. P	1.15
	Preferred	18.68	1.41	15.66	21.70			P vs. S	−2.67
	Slow	22.61	1.79	18.77	26.44			F vs. S	−1.14

* Differs from Preferred (*p* < 0.05);

** Differs from Preferred (*p* < 0.0001),

† Differs from Fast (*p* < 0.0001).
