# Supplementary material for: Margin of Stability May Be Larger and Less Variable during Treadmill Walking Versus Overground
Source: Biomechanics (Basel). Author manuscript; Available in PMC 2021 Aug 18. (PMC8372237; doi:10.3390/biomechanics1010009)
Supplement: Supplemental 2 [file NIHMS1700330-supplement-Supplemental_2.docx]

**SUPPLEMENTARY MATERIAL _ 2**

**Title:**

**Margin of stability is larger and less variable during treadmill walking versus overground**

**Authors:**

Farahnaz Fallahtafti^a^, Arash Mohammadzadeh Gonabadi^a,b^, Kaeli Samson^c^, Jennifer M. Yentes^a,d^

**Affiliations:**

^a^Department of Biomechanics, University of Nebraska at Omaha,

6160 University Drive South, Omaha, NE 68182-0860

^b^ Rehabilitation Engineering Center

Institute for Rehabilitation Science and Engineering

Madonna Rehabilitation Hospitals

5401 South Street, Lincoln, NE 68506, United states

^c^Department of Biostatistics, University of Nebraska Medical Center

984375 Nebraska Medical Center, Omaha, NE 68198-7259

^d^VA Nebraska-Western Iowa Health Care System, VA Research Service, 4104 Woolworth, Avenue (112), Omaha, NE 68105

**Corresponding Author:**

Farahnaz Fallahtafti

University of Nebraska at Omaha

6160 University Drive, Omaha, NE 68182-0860

Phone: 402.554.3225

Email: ffallahtafti@unomaha.edu

**Purpose:**

This supplementary material is provided to confirm the main effect of condition that was observed between Margin of Stability (MOS) on overground (OV) walking versus treadmill (TR) during preferred walking speed using Bayesian statistics.

**Methods:**

We used the data presented in a previous study [1], comparing the MOS in the mediolateral direction on overground versus treadmill walking at preferred walking speed to calculate the effect size that is needed to determine the Bayesian factor. All statistical tests were performed in JASP (JASP Team (2019). JASP (Version 0.11.1)).

$Cohen^{'}s d= \frac{M_{2}-M_{1}}{{SD}_{Pooled}}$ (1)

${SD}_{Pooled}=\sqrt{\frac{({SD}_{1}^{2}+{SD}_{2}^{2})}{2}}$ (2)

Table 1. Data of MOS from previous study [1].

|  | **Treadmill** | | **Overground** | |
| --- | --- | --- | --- | --- |
|  | **Left MOS** | **Right MOS** | **Left MOS** | **Right MOS** |
| **Mean (mm)** | 66.6 | 68.8 | 66.6 | 59.2 |
| **Standard Deviation (mm)** | 26.3 | 24.1 | 26.2 | 26.5 |
|  | **Treadmill** | | **Overground** | |
|  | **Combined Left and Right MOS** | | **Combined Left and Right MOS** | |
| **Mean (mm)** | 67.7 | | 62.9 | |
| **Standard Deviation (mm)** | 25.2 | | 26.35 | |

According to the results from combined left and right MOS for treadmill and overground in Table 1, the Cohen’s d was calculated (Cohen’s d = 0.18). We then ran the Bayesian paired sample T-test to evaluate the Bayesian factor (BF10) to confirm the mediolateral significant difference which was presented in the current study considering the small effect size. For the anterior-posterior direction we did not find the prior study to evaluate the effect size; therefore, we used default prior distribution (a two-tailed Cauchy distribution centered on zero with a scaling factor of 0.707) [2]. This default prior was used because small effects were more likely than large effects. We used Bayesian factor (BF01) to confirm the non-significant difference between walking modes in the anterior posterior direction during preferred walking speed.

**Results and Discussion**:

According to the results of the Bayesian analysis (Table 2), BF10 for MOS in the mediolateral direction, suggests that data is 2.539 times more likely to reject the null hypothesis rather than accepting the null hypothesis. According to the alternative hypothesis, MOS in the mediolateral direction during treadmill walking is larger than during overground walking. However, the BF01 for MOS in the anterior posterior direction indicates that chance of accepting the null hypothesis is 2.034 times greater than rejecting the null hypothesis, indicating that MOS in the anterior posterior direction between two walking modes is not different during preferred speed of walking.

Table 2. Bayesian Paired Sample T-test of the MOS in the mediolateral direction.

| **Measure 1** | **Measure 2** | **BF10** | **Error%** |
| --- | --- | --- | --- |
| Treadmill | Overground | 2.539 | 1.049e-5 |

Table 3. Bayesian Paired Sample T-test of the MOS in the anteroposterior direction.

| **Measure 1** | **Measure 2** | **BF01** | **Error%** |
| --- | --- | --- | --- |
| Treadmill | Overground | 2.034 | 5.229e-4 |

**References**

1. Rosenblatt, N.J. and M.D. Grabiner, *Measures of frontal plane stability during treadmill and overground walking.* Gait & Posture, 2010. **31**(3): p. 380-384.

2. Brydges, C.R. and A.A.M. Bielak, *A Bayesian Analysis of Evidence in Support of the Null Hypothesis in Gerontological Psychology (or Lack Thereof).* The Journals of Gerontology: Series B, 2020. **75**(1): p. 58-66.
