# Supplementary material for: Margin of Stability May Be Larger and Less Variable during Treadmill Walking Versus Overground
Source: Biomechanics (Basel). Author manuscript; Available in PMC 2021 Aug 18. (PMC8372237; doi:10.3390/biomechanics1010009)
Supplement: Supplemental 1 [file NIHMS1700330-supplement-Supplemental_1.docx]

**SUPPLEMENTARY MATERIAL**

**Title:**

**Margin of stability is larger and less variable during treadmill walking versus overground**

**Authors:**

Farahnaz Fallahtafti^a^, Arash Mohammadzadeh Gonabadi^a,b^, Kaeli Samson^c^, Jennifer M. Yentes^a,d^

**Affiliations:**

^a^Department of Biomechanics, University of Nebraska at Omaha,

6160 University Drive South, Omaha, NE 68182-0860

^b^ Rehabilitation Engineering Center

Institute for Rehabilitation Science and Engineering

Madonna Rehabilitation Hospitals

5401 South Street, Lincoln, NE 68506, United states

^c^Department of Biostatistics, University of Nebraska Medical Center

984375 Nebraska Medical Center, Omaha, NE 68198-7259

^d^VA Nebraska-Western Iowa Health Care System, VA Research Service, 4104 Woolworth, Avenue (112), Omaha, NE 68105

**Corresponding Author:**

Farahnaz Fallahtafti

University of Nebraska at Omaha

6160 University Drive, Omaha, NE 68182-0860

Phone: 402.554.3225

Email: ffallahtafti@unomaha.edu

**Purpose:**

This supplementary material is provided to identify the main effect of walking mode (treadmill vs. overground) and speed condition, while the steps were entered as separate values per subject per condition into an additional general estimating equation MOS AP model (instead of averaging steps within participant for each combination of speed and walking condition, as was done in the main manuscript). This allows for the assessment of the association between the variability in walking speed within a single pace condition (i.e. adjustments in speed to maintain target speed) and MOS AP.

**Methods:**

We prepared a long format data set including the 18 data points for MOS AP values at heel contact. The data set also included two additional values: 1) the target step speed for each pace (Fast, Preferred, and Slow), defined as the preferred walking speed as determined in the protocol; and 2) Speed-off, calculated as the difference between the step speed at the instant MOS was calculated and the target speed. Note that adjustments in speed (i.e. speed-off) were positive and negative in the overground condition, but only positive in the treadmill condition (i.e. participants did not walk slower than target speed on treadmill, presumably to avoid falling off). General estimating equation models were run for the right and left limbs separately. Models were run without covariates first and with speed-off as a covariate second.

**Results and Discussion**:

In models without any covariates, the difference between overground and treadmill MOS mean was only seen on the left side (p = 0.02). There was no difference in condition for the right limb. When speed-off was included as a covariate, both the right and the left MOS AP models, speed-off was significant (p = 0.004 and p < 0.0001, respectively) and therefore, no significant differences were seen between overground and treadmill conditions. This indicated that increases in deviations from target speed where associated with decreases in MOS AP. As can be seen in Supplemental Table 1, estimated means for MOS AP are very similar to those in Table 2 of the main manuscript. Similarly, the interactions between pace and condition are not significant, and the differences in MOS AP across speed conditions are significant between all combinations of speed conditions.

The main effect of condition was not significant for the right limb MOS AP, whether speed-off is included in the model or not. However, for the left limb MOS AP, the main effect of condition was not significant when speed-off was controlled in the model, but when speed-off was removed, it was significant. This may suggest that differences in MOS AP seen in the left measurements between treadmill and overground may be due to differences in correction in speed in each condition, and when we adjusted for the corrections in speed to maintain targeted speed (i.e. speed-off), this difference in walking conditions were disappeared. Therefore, differences in MOS AP between walking conditions may be due to the difference in speed adjustments that need to be made in two walking conditions. However, this should be explored further as the finding was demonstrated on one limb and not both.

If adjustment in speed from the target speed (i.e. speed-off) does lead to a difference between overground and treadmill walking, we might have expected to see a difference in walking condition in the main manuscript as speed-off was not included in the manuscript averaged models. Although, all manuscript averaged models adjusted for preferred walking speed as a covariate. This lack of difference between overground and treadmill mean MOS could be because data were averaged across left and right (and the right didn’t show this effect in the individual data model), or it could be due to the overall averaging, washing out this effect. As previously stated, this finding should be explored further as the differences were only seen in one limb when limbs were statistically separated.

**Supplemental Table 1. MOS AP Models for right and left limbs without and with speed-off covariate.**

| **Outcome/**  **Model** | **Pace and Condition Subgroups** | | **Model Estimated Mean** | | **Standard Error** | | **95% Confidence Interval** | | **P-value for differences within main effect** | **P-value for Interaction** |
| --- | --- | --- | --- | --- | --- | --- | --- | --- | --- | --- |
|  |  |  |  |  | |  | |  |  |  |
|  |  |  |  |  | |  | |  |  |  |
| **Right MOS AP: Without Speed-Off Covariate** | | | |  | |  | |  |  |  |
|  |  |  |  |  | |  | |  |  | 0.66 |
|  | **Pace** | |  |  | |  | |  | < 0.0001^ |  |
|  |  | Fast | -320.52 | 8.06 | | -337.81 | | -303.22 |  |  |
|  |  | Preferred | -170.21 | 8.06 | | -187.50 | | -152.91 |  |  |
|  |  | Slow | -32.95 | 8.06 | | -50.24 | | -15.66 |  |  |
|  | **Condition** | |  |  | |  | |  | 0.36 |  |
|  |  | OverGround | -173.18 | 7.94 | | -191.96 | | -154.40 |  |  |
|  |  | Treadmill | -175.93 | 7.94 | | -194.71 | | -157.15 |  |  |
|  |  |  |  |  | |  | |  |  |  |
|  |  |  |  |  | |  | |  |  |  |
|  |  |  |  |  | |  | |  |  |  |
| **Right MOS AP: With Speed-Off Covariate** | | | |  | |  | |  |  |  |
|  |  |  |  |  | |  | |  |  | 0.60 |
|  | **Pace** | |  |  | |  | |  | < 0.0001^ |  |
|  |  | Fast | -320.24 | 7.96 | | -337.31 | | -303.18 |  |  |
|  |  | Preferred | -169.95 | 7.96 | | -187.01 | | -152.88 |  |  |
|  |  | Slow | -33.48 | 7.96 | | -50.55 | | -16.41 |  |  |
|  | **Condition** | |  |  | |  | |  | 0.95 |  |
|  |  | OverGround | -174.66 | 7.85 | | -193.23 | | -156.10 |  |  |
|  |  | Treadmill | -174.45 | 7.85 | | -193.01 | | -155.89 |  |  |
|  | | | |  | |  | |  |  |  |
| **Left MOS AP: Without Speed-Off Covariate** | | | |  | |  | |  |  |  |
|  |  |  |  |  | |  | |  |  | 0.68 |
|  | **Pace** | |  |  | |  | |  | < 0.0001^ |  |
|  |  | Fast | -313.52 | 7.19 | | -328.95 | | -298.09 |  |  |
|  |  | Preferred | -165.55 | 7.19 | | -180.98 | | -150.12 |  |  |
|  |  | Slow | -34.20 | 7.19 | | -49.63 | | -18.78 |  |  |
|  | **Condition** | |  |  | |  | |  | 0.02 |  |
|  |  | OverGround | -167.23 | 7.07 | | -183.95 | | -150.50 |  |  |
|  |  | Treadmill | -174.96 | 7.07 | | -191.68 | | -158.23 |  |  |
|  | | | | | | | | | |  |
| **Left MOS AP: With Speed-Off Covariate** | | | | | | | | | |  |
|  |  |  |  |  | |  | |  |  | 0.58 |
|  | **Pace** | |  |  | |  | |  | < 0.0001^ |  |
|  |  | Fast | -313.11 | 7.13 | | -328.40 | | -297.81 |  |  |
|  |  | Preferred | -165.16 | 7.13 | | -180.45 | | -149.87 |  |  |
|  |  | Slow | -35.01 | 7.13 | | -50.31 | | -19.71 |  |  |
|  | **Condition** | |  |  | |  | |  | 0.28 |  |
|  |  | OverGround | -169.47 | 7.03 | | -186.09 | | -152.85 |  |  |
|  |  | Treadmill | -172.71 | 7.03 | | -189.33 | | -156.09 |  |  |
|  |  |  |  |  | |  | |  |  |  |
|  |  |  |  |  | |  | |  |  |  |
| All models adjust for preferred speed. Main effects are interpreted unless a significant interaction is present. | | | | | | | | | | |
| ^All pace conditions significantly differ from each other (p's < 0.0001). | | | | | | | |  |  |  |
